# Targeting Ninjurin‐1‐Mediated Microglial Pyroptosis Attenuates Neuroinflammation and Neuronal Injury Following Intracerebral Hemorrhage

**DOI:** 10.1002/brb3.71862

**Published:** 2026-11-01

**Authors:** Liqiang Peng, Aosheng Peng, Hui Zhu, Ting Sun, Pan Liu, Ting Qi

**Affiliations:** ^1^ Department of Trauma Center, Zhuzhou Hospital Affiliated to Xiangya School of Medicine Central South University Zhuzhou Hunan People's Republic of China; ^2^ Department of Emergency, Zhuzhou Hospital Affiliated to Xiangya School of Medicine Central South University Zhuzhou Hunan People's Republic of China; ^3^ Health Management Center, Zhuzhou Hospital Affiliated to Xiangya School of Medicine Central South University Zhuzhou Hunan People's Republic of China; ^4^ Department of General Surgery, Zhuzhou Hospital Affiliated to Xiangya School of Medicine Central South University Zhuzhou Hunan People's Republic of China

**Keywords:** intracerebral hemorrhage, microglia, NF‐κB, Ninjurin‐1, pyroptosis

## Abstract

**Objective**: This study aims to delineate the pathophysiological contribution of Ninjurin‐1 (Ninj1) to secondary neural injury following intracerebral hemorrhage (ICH), specifically elucidating its function in microglial pyroptosis and its upstream transcriptional governance by nuclear factor‐kappa B (NF‐κB).

**Methods**: Candidate targets were initially identified via in silico transcriptomic profiling. We subsequently employed a collagenase‐induced murine ICH paradigm alongside hemin‐challenged microglia. Mechanistic pathways were interrogated utilizing targeted genetic silencing (in vivo siRNA and in vitro shRNA) and pharmacological blockade. Disease phenotypes—encompassing pyroptotic execution, neuroinflammation, and collateral neuronal death—were quantified via immunoblotting, molecular assays, and histopathology. The upstream regulatory architecture was validated through ChIP‐qPCR, EMSA, and dual‐luciferase reporter assays.

**Results**: Bioinformatic screening highlighted Ninj1 as a prominently upregulated candidate transcript post‐ICH, a finding corroborated by robust induction in both in vivo and in vitro hemorrhagic models. Through prophylactic in vivo target validation, Ninj1 depletion restricted perihematomal edema and preserved sensorimotor function. Mechanistically, suppressing Ninj1 abrogated the microglial pyroptotic cascade and mitigated collateral neurotoxicity. Furthermore, NF‐κB was shown to transcriptionally activate NLRP3 and Ninj1, although direct promoter enrichment in the ChIP‐qPCR assay was statistically significant only for the Nlrp3 locus.

**Conclusions**: Ninj1 functions as a terminal executioner of microglial pyroptosis and a critical amplifier of post‐ICH neuroinflammation. Governed by a coordinated NF‐κB‐dependent transcriptional program alongside NLRP3, this signaling axis exacerbates secondary brain injury, positioning Ninj1 as a viable molecular candidate for neuroprotective target validation.

## Graphical abstract

In response to intracerebral hemorrhage, NF‐κB signaling upregulates Nlrp3 and Ninjurin‐1 (Ninj1) in microglia. NLRP3 inflammasome activation triggers GSDMD pore formation, whereas Ninj1 oligomerizes to execute terminal plasma membrane rupture (PMR) and DAMP release (such as HMGB1). Suppressing Ninj1 effectively prevents microglial pyroptosis and secondary neuroinflammation.

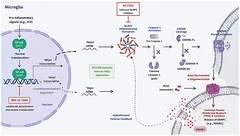

## 1 Introduction

As a severe subtype of hemorrhagic stroke, intracerebral hemorrhage (ICH) induces acute structural damage to the central nervous system (Li et al. 2025). Characterized by substantial rates of mortality, long‐term disability, and recurrence, this condition represents a major global public health burden (Gong et al. 2025). Contemporary clinical interventions predominantly rely on supportive measures, including blood pressure management, intracranial pressure reduction, and hematoma evacuation; however, the absence of targeted pharmacological therapies often results in unfavorable clinical outcomes (Chiang et al. 2025). The primary factor restricting therapeutic efficacy is secondary brain injury (SBI), which is precipitated by the hematoma and its associated toxic degradation products (Gao 2024). Consequently, elucidating the molecular mechanisms that propagate secondary neural damage is essential for the identification of novel neuroprotective targets.

During the progression of SBI, microglial activation serves as a central driver of the neuroinflammatory cascade (Zhou et al. 2026). Upon activation in the hemorrhagic microenvironment, microglia can undergo pyroptosis, a pro‐inflammatory programmed cell death. Distinct from apoptosis, this process involves plasma membrane rupture and the subsequent massive efflux of pro‐inflammatory cytokines, including IL‐1β, which exacerbates local tissue inflammation (Wei et al. 2022). The NOD‐like receptor family pyrin domain‐containing 3 (NLRP3) inflammasome acts as a critical mediator in this context. Post‐ICH NLRP3 activation actively propagates the inflammatory cascade; for example, it can be triggered by neutrophil extracellular traps (NETs) to promote pyroptosis and worsen neural injury (Zhang et al. 2025). Given its predominant expression in microglia, NLRP3 activation significantly amplifies neuroinflammatory signaling and neurological deficits (Xiao et al. 2023). Upstream of this complex, the NF‐κB signaling pathway serves as a canonical transcriptional activator for NLRP3, thereby aggravating inflammatory pathologies (Afonina et al. 2017). Despite the established role of the NLRP3 inflammasome in initiating microglial pyroptosis, the precise mechanisms governing its terminal downstream execution remain incompletely understood.

Ninjurin‐1 (Ninj1), initially characterized as a cell surface adhesion molecule, is classically implicated in tissue remodeling and neural regeneration following injury (Jennewein et al. 2015). For instance, previous studies have demonstrated its capacity to promote axonal growth and vascular recovery in models of diabetic neuropathy and spinal cord injury (Yin et al. 2014; Kim et al. 2021). Recently, however, its functional repertoire has expanded to include a critical role in the terminal execution phase of lytic cell death modalities. Specifically, Ninj1 has been identified as an essential mediator of plasma membrane rupture, triggering the extracellular release of damage‐associated molecular patterns (DAMPs) and amplifying inflammatory responses (Kayagaki et al. 2021). In the context of macrophage pyroptosis, Ninj1 orchestrates the final breakdown of the cell membrane subsequent to Gasdermin D (GSDMD) pore formation, a step strictly required for the extrusion of cellular contents (Degen et al. 2023). Nevertheless, its potential involvement in microglial pyroptosis during ICH, alongside the upstream transcriptional networks governing its expression, remains to be fully elucidated.

To address these knowledge gaps, the present study initially utilized transcriptomic profiling of public ICH datasets to identify candidate genes driving the acute neuroinflammatory response. Subsequently, employing both in vitro microglial models and an in vivo murine ICH paradigm, we delineated the specific contribution of Ninj1 to secondary neural injury. We sought to characterize its function as a terminal executor of microglial pyroptosis and to define its transcriptional regulation by the NF‐κB signaling axis. These findings aim to decode the complex microglia‐neuron crosstalk during ICH and evaluate Ninj1 as a viable molecular target for neuroprotective interventions.

## 2 Methods

### 2.1 Experimental Animals and ICH Model

Male C57BL/6 mice (8–10 weeks of age, weighing 22–25 g; *n = *96) were obtained from Vital River Company (Beijing, China). All animals were maintained in a specific pathogen‐free facility maintained at 22 ± 2°C and approximately 50% humidity, under a standard 12‐h light/dark circadian cycle.

To induce the ICH model, surgical anesthesia was initiated via 3% isoflurane inhalation and sustained with a 1% concentration. Following anesthetization, each animal was secured in a stereotaxic apparatus for preoperative preparation. A longitudinal incision was made along the midline of the scalp to expose the skull. Subsequently, a microinjection of 0.0375 U type IV collagenase (Sigma–Aldrich, C4‐28‐100MG), dissolved in 0.5 µL of sterile saline, was stereotaxically delivered into the right basal ganglia. The stereotaxic coordinates were set at 0.5 mm anterior, 2.3 mm lateral, and 3.5 mm ventral relative to the bregma. The injection was performed at a steady flow rate of 0.1 µL/min.

To prevent backflow, the microsyringe was left in situ for an additional 10 min prior to gradual withdrawal. The cranial burr hole was subsequently closed utilizing sterile bone wax, followed by surgical suturing of the scalp. Postoperative recovery was facilitated on a thermostatic heating pad (37°C). Control animals in the Sham group received equivalent surgical interventions, with the sole exception being the infusion of equal volumes of saline instead of collagenase (Deng et al. 2024).

At the designated 72‐h postoperative endpoint, euthanasia was performed by cervical dislocation following deep anesthesia with 1% sodium pentobarbital. Cerebral edema was quantitatively assessed via the standard wet‐dry weight technique. Briefly, the ipsilateral hemorrhagic hemisphere was rapidly excised and its initial mass recorded (wet weight). The samples were subsequently desiccated in an 80°C oven for 72 h to record the final mass (dry weight). Brain water content was calculated as: [(wet weight—dry weight) / wet weight] × 100%.

The in vivo experimental procedures obtained ethical clearance from the Animal Ethics Committee of Zhuzhou Hospital Affiliated to Xiangya School of Medicine (Approval No. K2025‐01‐223‐412). All protocols adhered strictly to institutional animal welfare regulations to guarantee humane treatment, and the study was reported in compliance with the ARRIVE guidelines.

### 2.2 In Vivo siRNA Administration

For in vivo gene silencing, specific small interfering RNA targeting Ninj1 alongside a scrambled negative control (NC) was obtained from GenePharma. Due to the proprietary nature of the commercial product, the exact nucleotide sequences remain undisclosed.

Forty‐eight hours preceding the ICH surgical procedure, subjects underwent anesthesia and were mounted on a stereotaxic apparatus. A microsyringe was positioned into the right lateral cerebral ventricle. The precise injection depth was controlled at 2.2–2.5 mm below the dura mater, utilizing the bregma as the anatomical zero point. A total payload of 2 nmol siRNA, dissolved in 4 µL of vehicle, was continuously infused at a controlled velocity of 0.2 µL/min. To avoid the reflux of the injected solution along the needle tract, the syringe was maintained in its original position for a 5‐min retention period prior to gradual removal.

To ensure objective evaluation, all subjects were allocated into designated experimental cohorts using a randomized approach. Appropriate sample sizes were prospectively calculated based on established literature and a priori power analyses, balancing the need for statistical robustness with ethical considerations for animal reduction. From the initial pool of 96 mice, 18 individuals were omitted from downstream evaluations secondary to intraoperative complications (such as insufficient or uncontrolled hemorrhage) or unanticipated mortality before the endpoint. This resulted in an aggregate exclusion and mortality rate of 18.75%.

A total of 78 mice successfully completed the protocol and were included in the final analyses. To comply with the ethical principle of animal reduction (3Rs) while maintaining statistical power, animals in each experimental group were assigned to two parallel cohorts. Cohort 1 (*n = *6 per group) was evaluated for sensorimotor functions (mNSS and Rotarod testing) and subsequently euthanized for brain water content determination. Cohort 2 (*n = *6 per group) was designated for terminal brain tissue analysis; within this cohort, three mice (*n = *3) were transcardially perfused with 4% paraformaldehyde for histological evaluations (Nissl and immunofluorescence staining), and the remaining three mice (*n = *3) were euthanized without perfusion to collect fresh‐frozen perihematomal tissues for molecular assays (qRT‐PCR, ELISA, and Western blot). To ensure objectivity, investigators performing behavioral grading and histological quantifications were blinded to the experimental group assignments.

### 2.3 Cell Culture and In Vitro Models

The immortalized BV‐2 microglial and HT22 neuronal cell lines (SCSP‐5208, Cell Bank of the Chinese Academy of Sciences; GCL‐0697, Wuhan Pricella) were selected for preliminary in vitro assays due to their stable transfection profiles. These cells were cultured conventionally in Dulbecco's Modified Eagle medium (DMEM) supplemented with 10% fetal bovine serum (FBS) and 1% penicillin/streptomycin, kept at 37°C in a humidified 5% CO_2_ incubator.

To construct the in vitro hemorrhage model, microglia were incubated with hemin (100 µM; Sigma–Aldrich) for 24 h. For targeted pathway inhibition, cultures were pretreated with either MCC950 (1 µM) or BAY 11‐7082 (5 µM; MCE) for 1 h prior to hemin administration. In genetic manipulation experiments, cells were transfected with sh‐Ninj1 alone or co‐transfected with an oe‐Ninj1 plasmid using the Lipofectamine 3000 reagent (Invitrogen) before hemin treatment.

Furthermore, primary microglia were isolated from the cerebral cortices of postnatal Day 1–3 (P1‐P3) C57BL/6 mice to validate the immortalized cell data. Following enzymatic digestion, the resultant mixed glial cells were maintained in DMEM/F12 for two weeks. Microglial populations were subsequently purified through mild orbital shaking and collected for downstream analysis.

### 2.4 RNA Sequencing Data Processing and Bioinformatics Analysis

Transcriptomic datasets (GSE290311 and GSE228222) were retrieved from the Gene Expression Omnibus (GEO) repository. Initial data quality was verified through principal component analysis (PCA) applied to each dataset independently. Following the consolidation of normalized expression matrices for shared genes, differential expression evaluation was executed via the limma R package.

To mitigate potential technical bias during dataset integration, the study cohort source was designated as a batch covariate within the limma linear model design matrix prior to the application of empirical Bayes moderation. This procedure effectively isolates authentic biological variance (ICH vs. sham) from systemic artifacts. Statistical thresholds for identifying differentially expressed genes (DEGs) were strictly defined as an adjusted *p*‐value < 0.05 alongside an absolute log2 fold change > 2. Downstream functional annotations, including gene ontology (GO) and Kyoto Encyclopedia of Genes and Genomes (KEGG) pathways, were mapped for the upregulated DEGs employing the clusterProfiler package

### 2.5 ELISA

The concentration of IL‐1β was quantified employing a commercial enzyme‐linked immunosorbent assay (ELISA) kit (abcam, ab197742) in strict adherence to the manufacturer's instructions. Following the establishment of appropriate blank and standard controls, the experimental samples were subjected to sequential incubations with biotinylated and enzyme‐conjugated antibodies. Upon the introduction of a colorimetric substrate and subsequent reaction termination, the optical density (OD) was assessed at a wavelength of 450 nm via a Bio‐Rad 680 microplate reader (Bio‐Rad, USA).

### 2.6 Immunofluorescence (IF)

Following appropriate interventions, coverslip‐adherent BV‐2 microglia underwent fixation in 4% paraformaldehyde (15 min) and subsequent permeabilization using 0.2% Triton X‐100 (10 min) at ambient temperature. Non‐specific binding sites were masked by applying 5% bovine serum albumin (BSA) for 1 h. The preparations were then exposed to a primary antibody cocktail targeting Ninj1 (abcam, ab325137) and IBA1 (Boster, ab283319) overnight at 4°C.

This was followed by a 60‐min incubation at 37°C with corresponding fluorophore‐conjugated secondary antibodies (Alexa Fluor 488, abcam, ab150081; Alexa Fluor 594, abcam, ab150116). Cellular nuclei were visualized via DAPI counterstaining. Finally, the slides were sealed with an anti‐fade mounting reagent, and fluorescent signals were captured utilizing a CSU‐X1 confocal laser scanning microscope (Nikon, Japan).

### 2.7 TUNEL Staining

In order to evaluate apoptotic cell death among neurons adjacent to the hematoma, brain sections were processed using an in situ cell death detection kit (Beyotime, C1089). Following routine deparaffinization, hydration, and permeabilization with proteinase K, the tissue slices were exposed to the TUNEL labeling mix in a dark, humid environment at 37°C for 1 h. After thorough washing steps, the specimens were subjected to standard immunofluorescence protocols targeting the neuronal marker NeuN (detailed previously). Nuclear architecture was highlighted using DAPI. The relative frequency of double‐positive (TUNEL+/NeuN+) cells was subsequently determined via fluorescence microscopy.

### 2.8 Nissl Staining

The extent of neuronal damage was evaluated through Nissl histochemistry (Beyotime, C0117). Tissue slices were first cleared in xylene and rehydrated via descending ethanol gradients. Subsequent staining allowed for the morphological assessment of surviving versus degenerating neurons within the affected perihematomal region. Micrographs were acquired utilizing a standard light microscope. To quantify neuronal survival, five randomly selected, non‐overlapping fields of view at 400× magnification within the perihematomal zone were examined per brain slice (representing three animals per experimental group). Viable neurons, characterized by a well‐defined cell body containing a clear, round nucleus with a distinct nucleolus, were enumerated using ImageJ software by an investigator blinded to the experimental groups.

### 2.9 Lactate Dehydrogenase (LDH) Release Assay

Plasma membrane integrity was evaluated by quantifying LDH release with a colorimetric Cytotoxicity Detection Kit (Beyotime, C0016). Culture media were harvested and centrifuged to remove cellular debris. The clarified supernatant was reacted with the supplied working solution for 5 min, after which the optical density at 490 nm was recorded on a Bio‐Rad 680 microplate reader (Bio‐Rad, USA).

### 2.10 Flow Cytometry for Apoptosis Detection

The apoptotic fraction of neuronal cultures was determined utilizing an Annexin V‐FITC/PI Apoptosis Detection Kit (ServiceBio, G1511). Following appropriate treatments, cells were detached using trypsin, washed in buffered saline, and double‐stained with the provided fluorochromes. The labeled populations were immediately analyzed utilizing a BD FACSCalibur flow cytometer to establish the percentage of apoptotic events.

### 2.11 Quantitative Real‐Time PCR (qRT‐PCR)

For transcript quantification, cellular RNA was isolated employing TRIzol Reagent (Beyotime, R0016). Following this, 1 µg of the purified RNA template underwent reverse transcription to generate cDNA via a Reverse Transcription Kit (Beyotime, D7170). Quantitative PCR was subsequently executed on a Bio‐Rad CFX96 Real‐Time PCR system, incorporating SYBR Green Master Mix (Beyotime, D7260) and custom oligonucleotides. The specific sequences utilized for amplification are detailed in Table 1.

**TABLE 1:** The sequences of all primers.

Gene	Sequence (5'–>3')
Ninj1‐forward	ACTGAGGAGTATGAGCTCA
Ninj1‐reverse	TCCATTACAGGCTTCTGGA
GAPDH‐forward	GGAGCGAGATCCCTCCAAAAT
GAPDH‐reverse	GGCTGTTGTCATACTTCTCATGG

### 2.12 Western Blot Analysis

Total cellular proteins were extracted using RIPA buffer (Merck, 20‐188), and the resulting lysates were clarified by centrifugation prior to concentration assessment. Equal amounts of protein samples were resolved via 10% SDS‐PAGE and electroblotted onto PVDF membranes. Non‐specific sites were saturated with 5% non‐fat milk, followed by a 4°C overnight incubation with specific primary antibodies targeting Ninj1 (abcam, ab325137), cleaved caspase‐1 (Cell Signaling Technology, 4199T), cleaved GSDMD (abcam, ab215203), Cleaved Caspase‐3 (Cell Signaling Technology, 9661T), NLRP3 (Boster, BA3677), and GAPDH (Boster, BM3874). The blots were subsequently probed with HRP‐conjugated secondary antibodies (abcam, ab97051) for 1 h at ambient temperature. Immunoreactive bands were ultimately developed utilizing an ECL kit (Proteintech, PK10003).

### 2.13 Electrophoretic Mobility Shift Assay (EMSA) and Supershift Assay

Protein‐DNA interactions between the transcription factor NF‐κB (p65) and the Nlrp3 and Ninj1 promoter regions were assessed employing a chemiluminescent EMSA kit (Thermo Fisher Scientific, 20148X). Biotinylated oligonucleotides encompassing the predicted NF‐κB response elements were synthesized by GenePharma (Shanghai, China). These labeled probes were mixed with nuclear fractions derived from hemin‐stimulated BV‐2 cells. For specificity and supershift validations, parallel binding reactions were supplemented with an anti‐p65 antibody (Boster, A00284‐1), excess unlabeled competitor, or mutant probes. The assembled nucleoprotein complexes were resolved on an 8% polyacrylamide gel. Following transfer, the complexes were visualized through incubation with a streptavidin–horseradish peroxidase conjugate coupled with chemiluminescent detection.

### 2.14 Chromatin Immunoprecipitation (ChIP)‐qPCR Assay

To evaluate the physical interaction between the NF‐κB (p65) transcription factor and the promoter regions of NLRP3 and Ninj1, chromatin immunoprecipitation was executed utilizing a ChIP Assay Kit (Beyotime, P2078). Following endogenous NF‐κB activation via hemin treatment, BV‐2 cells were subjected to cross‐linking with 1% formaldehyde (10 min, ambient temperature) prior to DNA fragmentation via sonication. The resulting chromatin fragments were immunoprecipitated overnight using protein A/G magnetic beads pre‐coated with either an anti‐p65 antibody (Boster, A00284‐1) or a control IgG antibody (abcam, ab109489). Subsequent to sequential washing and reverse cross‐linking, the recovered DNA fragments were purified. Finally, qPCR was employed to quantify the relative enrichment of the NLRP3 and Ninj1 promoter sequences within the immunoprecipitates.

### 2.15 Dual‐Luciferase Reporter Assay

Transcriptional regulatory activities were assessed in HEK293T cells cultured in 96‐well plates (50%–70% confluence). Transfection complexes were formulated utilizing the Lipo8000 transfection reagent (Beyotime, C0533). Specifically, reporter plasmids containing either wild‐type or mutated promoter sequences (WT/MUT‐NLRP3‐Luc or WT/MUT‐Ninj1‐Luc) were co‐transfected with an NF‐κB (p65) overexpression plasmid (or its corresponding empty vector, EV) and the pRL‐TK internal control plasmid. The lipid‐DNA mixtures were incubated at room temperature for 20 min before being applied to the cells in fresh culture medium. Following a 48 h incubation at 37°C under 5% CO_2_, cellular lysates were collected. Bioluminescent signals corresponding to firefly (*Photinus pyralis*) and Renilla (*Renilla reniformis*) luciferases were quantified utilizing a multifunctional microplate reader. Transcriptional activity was expressed as the normalized ratio of firefly to Renilla luminescence.

### 2.16 Co‐Culture

A non‐contact co‐culture system was established utilizing Transwell inserts to evaluate microglial‐neuronal crosstalk. BV‐2 cells were seeded into the apical chambers at a density of 2×10^5^ cells per well, while target neuronal cells were plated in the basolateral compartments. Both cell populations were initially maintained independently in standard DMEM supplemented with 10% FBS at 37°C under 5% CO_2_. Upon reaching optimal confluence, the microglial inserts were transferred into the neuronal plates, allowing for the exchange of soluble mediators. The combined system was then co‐incubated for an additional 24 h prior to downstream evaluations.

### 2.17 Rotarod Test

To evaluate post‐injury motor coordination, the accelerated Rotarod paradigm was employed. Prior to surgical induction of ICH, subjects were acclimated to the apparatus over a 3‐day consecutive training period. The conditioning protocol consisted of dual daily sessions, during which the rotational speed uniformly accelerated from 4 to 40 rpm over a maximal duration of 5 min (300 s). At the designated 72 h post‐ICH evaluation point, the tested subjects underwent three independent trials daily, and the mean latency to fall was calculated for downstream analyses.

### 2.18 Neurological Function Assessment

Postoperative neurological deficits were quantified utilizing the modified Neurological Severity Score (mNSS) system. This comprehensive behavioral rubric integrates evaluations of motor function (e.g., muscle tone, gait), sensory function (vision, touch), balance, and reflexes. The scoring spectrum ranges from 0 to 18, categorically defining discrete levels of impairment (0, normal; 1–6, mild injury; 7–12, moderate injury; and 13–18, severe injury), with ascending numerical values corresponding to exacerbated neurological dysfunction.

### 2.19 Statistical Analysis

Quantitative outcomes are expressed as the mean ± standard deviation (SD). All analytical procedures were executed within the GraphPad Prism 8.0 environment. For dual‐group comparisons (such as qRT‐PCR in Figure 2A and ChIP‐qPCR enrichment assays in Figure 6B), an unpaired Student's *t*‐test was applied, with Welch's correction incorporated where variance inequality was detected. For comparisons involving three or more experimental groups (such as Figures 3, 4, 5 and 7), one‐way analysis of variance (ANOVA) followed by Tukey's post‐hoc test was performed. Statistical significance was predefined at *p < *0.05, and exact *p* values for key statistical comparisons are reported in the respective figure legends and text.

## 3 Results

### 3.1 Bioinformatic Identification of Candidate Target Molecules and Signaling Pathways in ICH

To elucidate the molecular mechanisms underlying post‐ICH neural injury, we conducted an integrated re‐analysis of publicly available transcriptomic datasets derived from murine ICH models. Initial quality control via PCA demonstrated that the samples segregated primarily based on biological conditions (ICH vs. sham) along PC1, whereas dataset‐specific batch variations were separated along PC2 (Figure 1A). This distribution pattern confirmed a robust disease‐associated transcriptional signature alongside mathematically correctable batch effects. Following the verification of data normalization through uniform expression distributions (Figure 1B) and subsequent batch correction, differential expression analysis was executed. By applying rigorous screening thresholds (|log2FoldChange| > 2, *p*_adj_ < 0.05), a substantial panel of differentially expressed genes (DEGs) was isolated, as depicted in the volcano plot (Figure 1C). Notably, Ninj1—a molecule closely associated with the execution of plasma membrane rupture—emerged as one of the most prominently upregulated candidate genes. Subsequent functional enrichment profiling of these upregulated DEGs strongly indicated the activation of an acute immunological cascade. Specifically, GO annotations were significantly enriched for terms including “myeloid leukocyte activation”, “chemotaxis”, and “phagocytosis” (Figure 1D), while KEGG pathway mapping correspondingly highlighted “phagosome”, “lysosome”, and multiple inflammation‐related signaling networks (Figure 1E). Collectively, this bioinformatic screening of bulk brain tissue strongly implicated Ninj1 as a critical candidate within the post‐hemorrhagic inflammatory response, thereby prompting subsequent experimental investigations into its specific cellular origin and functional role.

**FIGURE 1:**
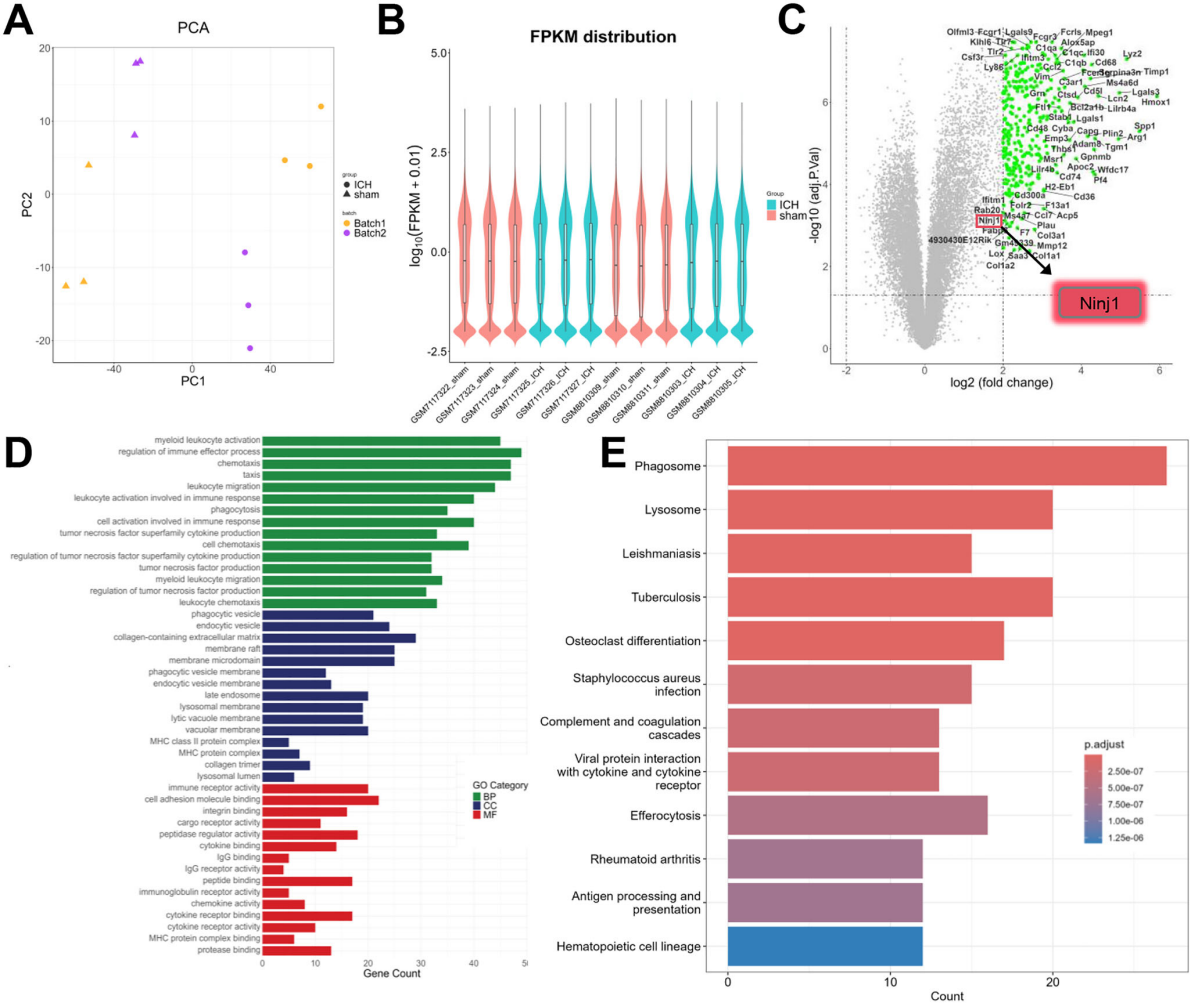
Bioinformatic identification of candidate target molecules and signaling pathways in ICH. (A) PCA showed sample distribution. (B) Gene expression distribution was illustrated by violin plots. (C) Volcano plot showing differentially expressed genes (DEGs) in microglia, with Ninj1 highlighted. (D) Gene ontology (GO) functional enrichment analysis of upregulated DEGs. (E) Kyoto Encyclopedia of Genes and Genomes (KEGG) pathway analysis of upregulated DEGs.

### 3.2 Ninj1 is Highly Expressed in Microglia After Intracerebral Hemorrhage

To evaluate the pathophysiological involvement of Ninj1 during hemorrhagic injury, a standardized in vivo murine ICH model was employed. Subsequent molecular assessments at 72 h post‐injury via qRT‐PCR and immunoblotting demonstrated a robust elevation in both Ninj1 transcript and protein expression within the perihematomal microenvironment relative to sham‐operated controls (Figure 2A,B). To elucidate the specific spatial distribution of this upregulated signal, dual‐immunofluorescence labeling was conducted. Confocal imaging, complemented by quantitative fluorescence profile analysis, revealed a striking spatial co‐localization between Ninj1 (red fluorescence) and the canonical microglial marker IBA1 (green fluorescence) (Figure 2C). Furthermore, to bioinformatically substantiate this precise cellular origin across the broader hemorrhagic landscape, an independent public single‐cell RNA sequencing (scRNA‐seq) dataset (GSE282308) was interrogated. Single‐cell transcriptomic profiles (UMAP and violin plots) indicated that Ninj1 was enriched across myeloid‐lineage populations, showing prominent transcript levels in neutrophils, activated macrophages, and microglia/macrophages, whereas expression in resident non‐myeloid central nervous system cells remained negligible (Figure S3). Collectively, this integrated in vivo and in silico evidence demonstrates that Ninj1 is robustly induced and predominantly localized within microglia following ICH.

**FIGURE 2:**
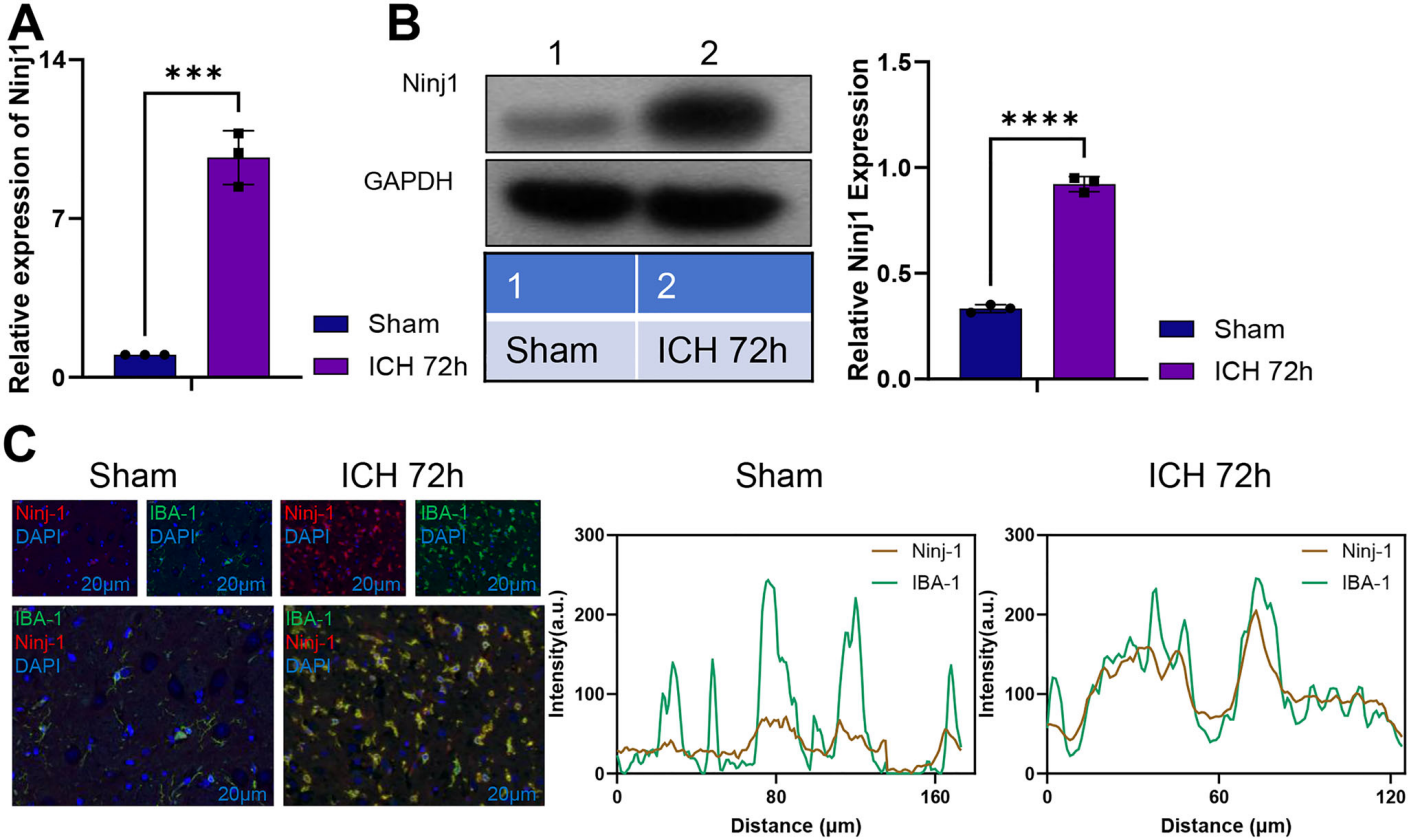
Ninj1 expression in perihematomal brain tissues after murine ICH. (A) qRT‐PCR analysis of Ninj1 mRNA (*n = *3). (B) Representative immunoblots and relative quantification of Ninj1 protein (*n = *3). (C) Double immunofluorescence of Ninj1 (red) and IBA1 (green) with nuclear counterstain (DAPI, blue) and corresponding spatial intensity profile scans (scale bar = 20 µm, *n = *3). Data represent mean ± SD. While behavioral and edema assessments utilized the full cohort (*n = *6 per group), molecular and histological assays (A–C) were performed on partitioned tissue subsets (*n = *3 per group). Significant differences were determined by unpaired Student's *t*‐test (with Welch's correction applied for Figure 2A, exact *p = *0.0002; for Figure 2B, *p < *0.0001; **p < 0.001, *p < 0.0001).

### 3.3 Ninj1 Mediates Hemin‐Induced Microglial Pyroptosis

To elucidate the specific function of Ninj1 in microglial pyroptosis, shRNA‐mediated silencing was performed in hemin‐challenged BV‐2 cells. Upon verifying target depletion (Figure 3A), canonical pyroptotic mediators were assessed. Immunoblotting demonstrated that Ninj1 suppression concurrently diminished the proteolytic maturation of caspase‐1 and GSDMD (Figure 3B). Consequently, plasma membrane integrity was preserved and inflammatory signaling was restricted, as evidenced by reduced LDH and IL‐1β efflux (Figure 3C,D). To ascertain the mechanistic hierarchy, a gain‐of‐function rescue assay was implemented. Ectopic re‐introduction of Ninj1 (oe‐Ninj1) effectively reversed the sh‐Ninj1‐induced suppression of upstream caspase‐1 and GSDMD cleavage (Figure S1), supporting the hypothesis of a positive feedback loop that modulates upstream inflammasome activation. Furthermore, to exclude artifacts inherent to immortalized cell cultures, these core molecular dynamics were corroborated in primary murine microglia. Hemin‐induced Ninj1 elevation and the associated Caspase‐1/GSDMD processing were consistently abrogated by targeted Ninj1 knockdown in this primary system (Figure S2). Collectively, these observations position Ninj1 as both a terminal executioner and an active amplifier of the microglial pyroptotic cascade.

**FIGURE 3:**
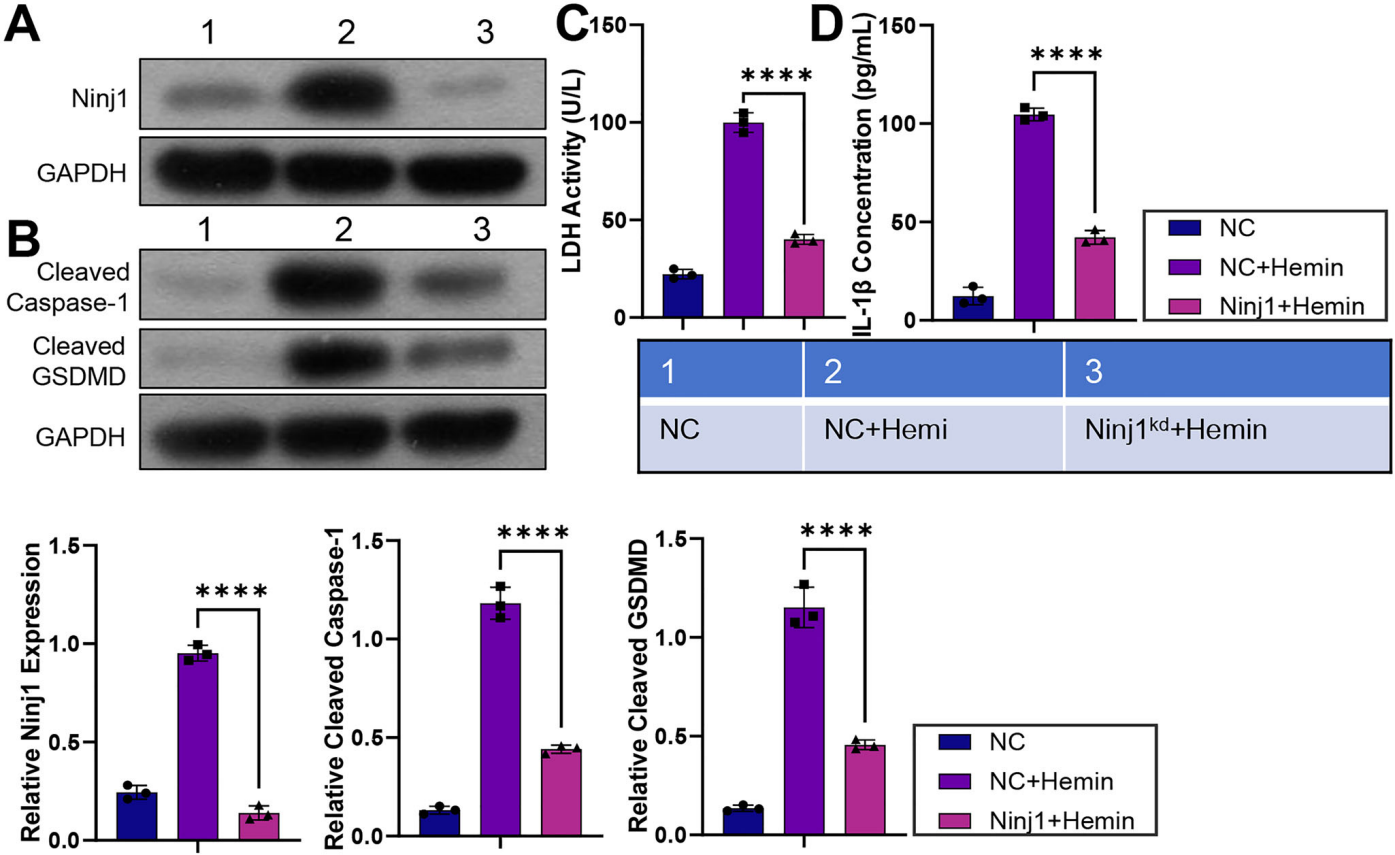
Ninj1 mediates hemin‐induced microglial pyroptosis. (A) The knockdown efficiency of Ninj1 shRNA was validated by Western blot. (B) Protein levels of pyroptosis‐related proteins (Cleaved Caspase‐1, Cleaved GSDMD) were detected by Western blot. (C) LDH release was measured to assess plasma membrane rupture. (D) The secretion of IL‐1β was quantified by ELISA. Data are presented as mean ± SD, *n = *3 independent experiments. *p < 0.05, **p < 0.01, ***p < 0.001, ****p < 0.0001.

### 3.4 Knockdown of Ninj1 Alleviates Microglial Pyroptosis‐Mediated Neuronal Injury

To evaluate the paracrine neurotoxicity elicited by microglial pyroptosis, neurons were cultured in conditioned medium (CM) derived from hemin‐challenged BV‐2 cells subjected to targeted Ninj1 silencing. Subsequent flow cytometric and immunoblotting assessments revealed that CM from these Ninj1‐depleted microglia effectively mitigated the overall neuronal apoptosis rate while concurrently diminishing the activation of cleaved caspase‐3 (Figure 4A,B). These observations confirm that Ninj1‐dependent microglial pyroptosis functions as a critical driver of collateral secondary neuronal damage.

**FIGURE 4:**
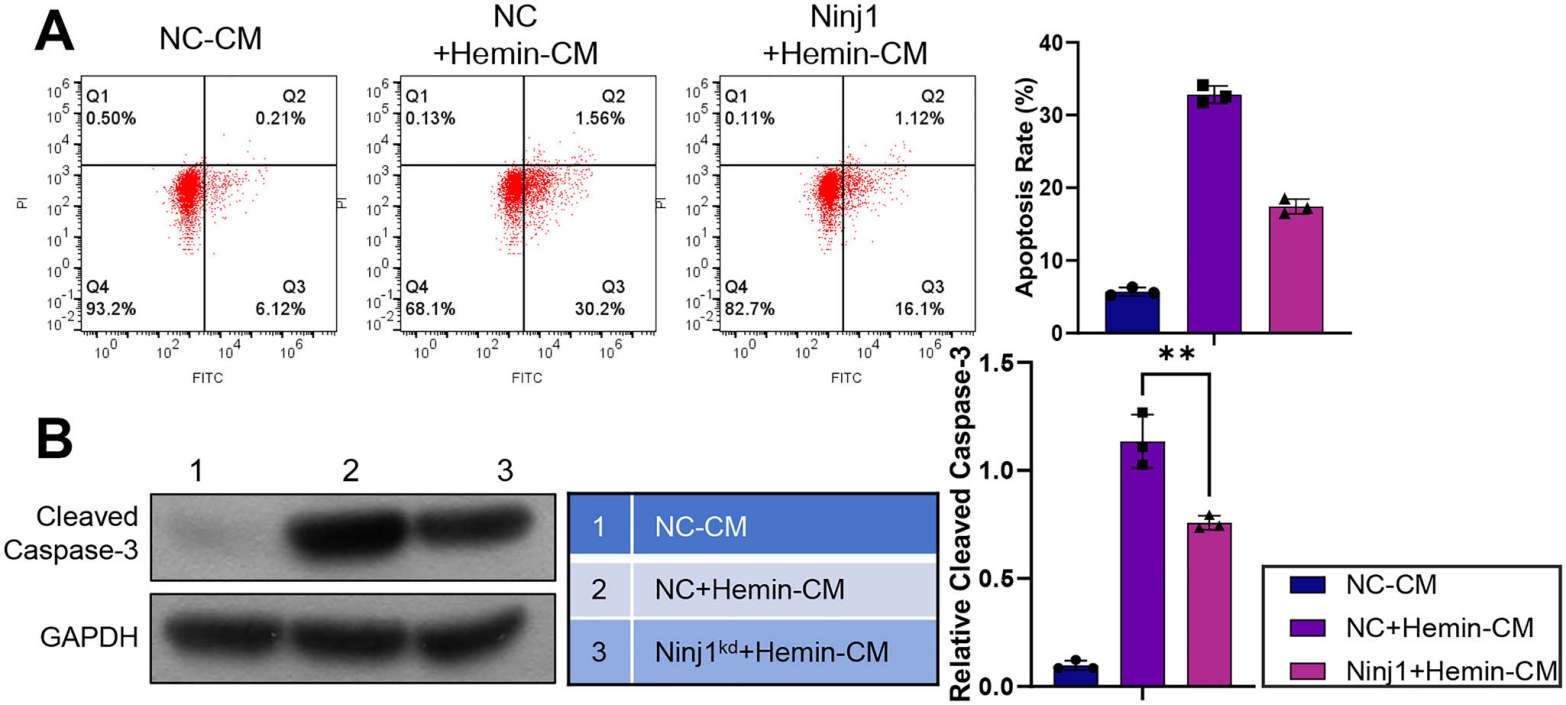
Knockdown of Ninj1 alleviates microglial pyroptosis‐mediated neuronal injury. (A) Neuronal apoptosis was detected by flow cytometry. (B) The level of the apoptotic protein Cleaved Caspase‐3 in neurons was detected by Western blot. Data are presented as mean ± SD, *n = *3 independent experiments. *p < 0.05, **p < 0.01.

### 3.5 Ninj1 Induction is Only Partially Dependent on NLRP3 Inflammasome Activation

To delineate the hierarchical dynamics between Ninj1 and the canonical NLRP3 inflammasome cascade, hemin‐challenged microglia were pre‐incubated with the selective NLRP3 antagonist MCC950. Subsequent LDH efflux assessments verified that this pharmacological blockade blunted the overall pyroptotic response (Figure 5A). Immunoblotting corroborated this effect by revealing diminished proteolytic maturation of caspase‐1; importantly, however, while MCC950 treatment resulted in a partial but statistically significant reduction in hemin‐induced Ninj1 protein levels, Ninj1 expression remained significantly elevated compared with the control group (Figure 5B). This partial downregulation suggests that although the initial induction of Ninj1 occurs independently of NLRP3 activation, its sustained upregulation may be further amplified through a hypothesized positive feedback loop driven by lytic cell death and subsequent extracellular inflammatory signaling. In parallel co‐culture paradigms, conditioned medium (CM) derived from these MCC950‐treated microglia conferred robust neuroprotection, restricting neuronal Cleaved Caspase‐3 activation and reducing overall apoptosis rates compared to hemin‐CM controls (Figure 5B,C). Collectively, these data demonstrate that Ninj1 induction is not a downstream consequence of NLRP3 activation, suggesting a potentially coordinated upstream regulatory framework governing the microglial pyroptotic program.

**FIGURE 5:**
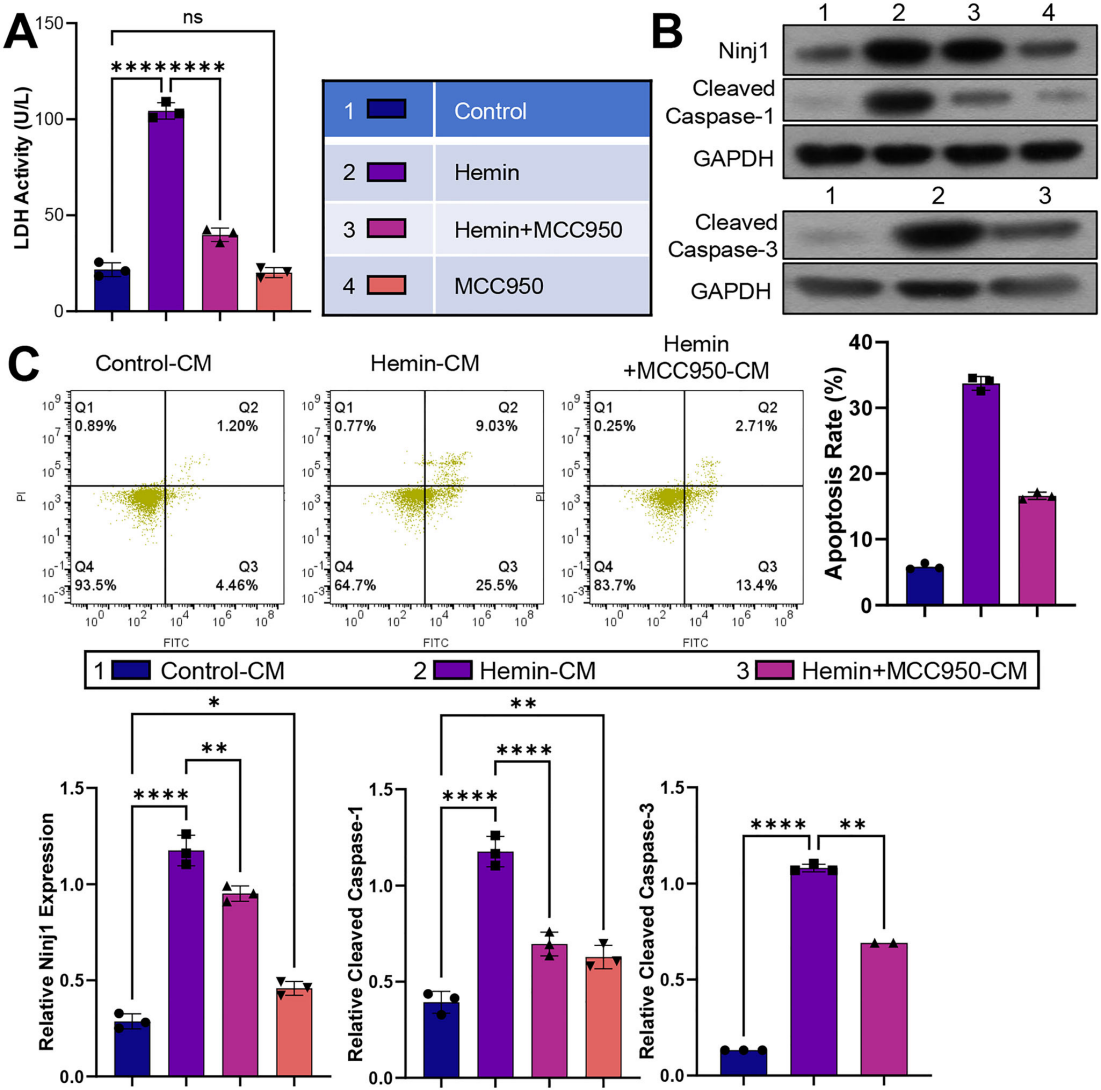
Ninj1 induction is only partially dependent on NLRP3 inflammasome activation. (A) The effect of the NLRP3 inhibitor MCC950 on LDH release was measured. (B) Protein levels of Ninj1, Cleaved Caspase‐1 in microglia, and Cleaved Caspase‐3 in co‐cultured neurons were detected by Western blot. (C) Neuronal apoptosis was detected by flow cytometry. Data are presented as mean ± SD, *n = *3 independent experiments. *p < 0.05, **p < 0.01, ***p < 0.001, ****p < 0.0001; ns = non‐significant.

### 3.6 NF‐κB Coordinates the Transcriptional Activation of Ninj1 and NLRP3

Building upon the observation that Ninj1 induction is uncoupled from downstream inflammasome activation, we hypothesized a shared upstream transcriptional framework, leading us to investigate the canonical NF‐κB cascade. Initial dual‐luciferase reporter assays demonstrated that ectopic NF‐κB expression amplified the luminescence of constructs harboring the wild‐type Ninj1 and NLRP3 promoter regions (Figure 6A). Subsequent physical interaction analyses via ChIP‐qPCR confirmed the substantial enrichment of the NF‐κB p65 subunit at the Nlrp3 promoter locus within BV‐2 cells, whereas p65 enrichment at the Ninj1 promoter did not reach statistical significance under these conditions (Figure 6B). This direct protein‐DNA engagement was further corroborated by EMSA‐supershift assays, which exhibited specific p65 binding to the respective promoter probes (Figure 6C). Translating these findings to the hemin‐challenged in vitro model, immunofluorescence tracking illustrated enhanced p65 nuclear translocation, a process selectively restricted by the NF‐κB antagonist BAY 11–7082 (Figure 6D). Correspondingly, immunoblot evaluations verified that pharmacological blockade with BAY 11‐7082 blunted the concurrent hemin‐induced elevation of both Ninj1 and NLRP3 proteins (Figure 6E). Together, these multidimensional data indicate that NF‐κB regulates the expression of both Nlrp3 and Ninj1, though the direct chromatin‐binding profiles of p65 differ between these target promoters.

**FIGURE 6:**
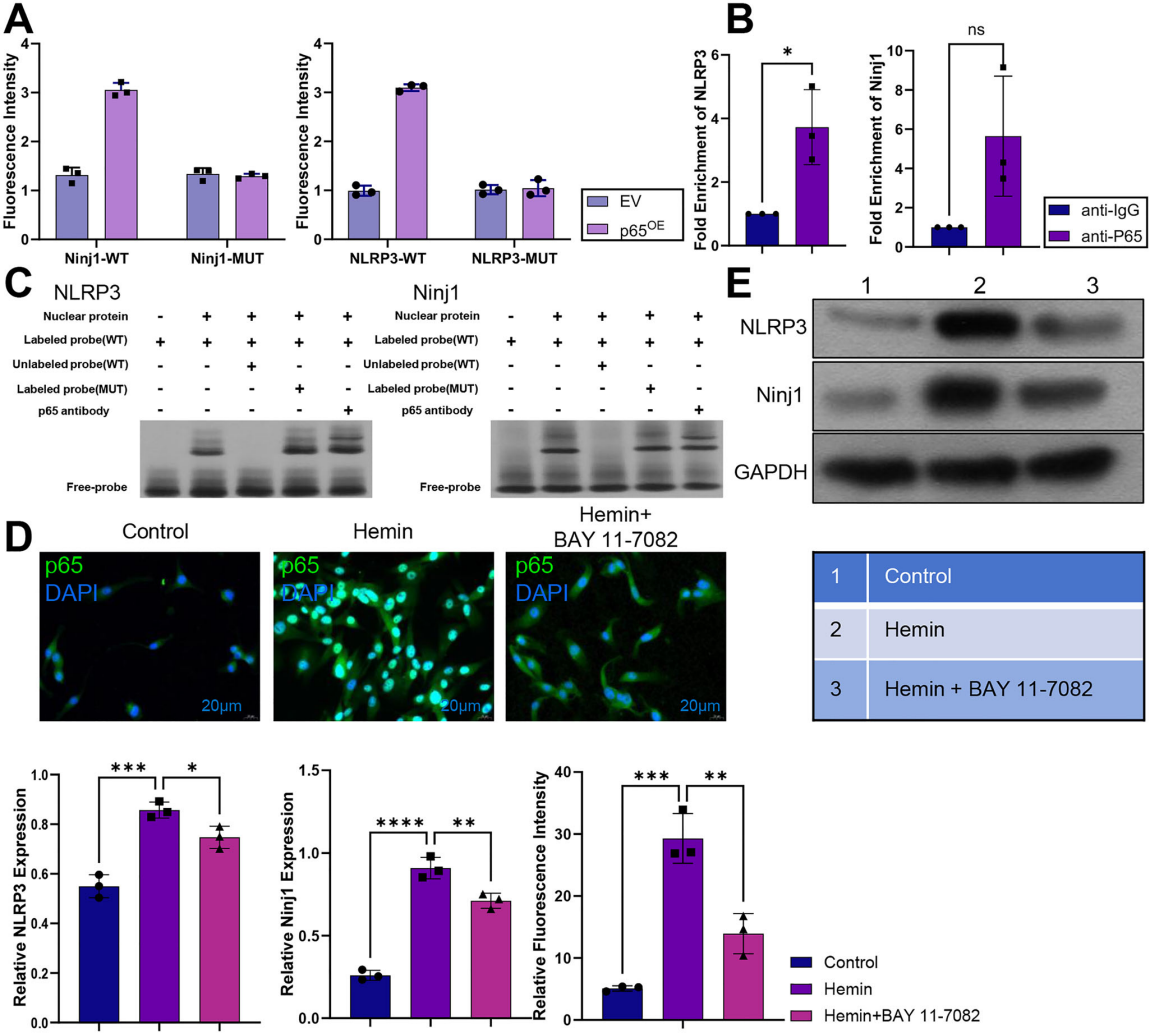
NF‐κB coordinates the transcriptional activation of Ninj1 and NLRP3. (A) Dual‐luciferase reporter assay was performed to verify the transcriptional regulation of Ninj1 and NLRP3 by NF‐κB. (B) ChIP‐qPCR assay assessing the fold enrichment of NF‐κB p65 at the promoter regions of Nlrp3 (exact *p = *0.0161, unpaired Student's t‐test) and Ninj1 (exact *p = *0.0582, ns = non‐significant, unpaired Student's t‐test). (C) EMSA and supershift assay confirming the specific binding of NF‐κB p65 to the promoters of Ninj1 and NLRP3. (D) Nuclear translocation of NF‐κB p65 was observed by immunofluorescence. (E) The effect of the NF‐κB inhibitor BAY 11–7082 on Ninj1 and NLRP3 protein levels was detected by Western blot. Scale bar = 20 µm. Data are presented as mean ± SD, *n = *3 independent experiments. *p < 0.05, **p < 0.01, ***p < 0.001, ****p < 0.0001; ns = non‐significant.

### 3.7 In Vivo Target Validation of Ninj1 Confirms Its Role in Post‐ICH Neurological Injury

To provide rigorous in vivo target validation, prophylactic intracerebroventricular administration of Ninj1‐targeting siRNA (Ninj1kd) or control siRNA (ICH) was executed 48 h prior to ICH induction. Subsequent behavioral evaluations revealed that while the ICH cohort exhibited severe sensorimotor deficits—reflected by elevated mNSS scores and diminished rotarod endurance—Ninj1 knockdown significantly preserved overall neurological function and motor coordination (Figure 7A,B). Corresponding histopathological assessments demonstrated that this prophylactic depletion restricted the accumulation of perihematomal edema (Figure 7C), limited the excessive tissue accumulation of IL‐1β (Figure 7D), and rescued local neuronal populations, as verified by a significant recovery in surviving neuron density (135 ± 12 vs. 78 ± 10 cells/HPF, Figure 7E), compared to control‐injected animals. At the molecular level, immunoblotting confirmed that the pronounced post‐hemorrhagic upregulation of total Ninj1 and cleaved caspase‐1 was blunted by this targeted genetic intervention (Figure 7F). Complementary immunofluorescence mapping further visualized this effect, illustrating that the profound induction of both Ninj1 and the terminal executioner Cleaved GSDMD within IBA1‐positive myeloid cells (encompassing microglia and macrophages) was distinctly abrogated following in vivo Ninj1 knockdown (Figure 7G,H). Collectively, these target validation data indicate that suppressing myeloid‐lineage Ninj1 curtails the broader neuroinflammatory cascade, minimizes collateral neuronal loss, and supports functional neurological recovery following ICH, representing a non‐cell‐specific target validation of Ninj1.

**FIGURE 7:**
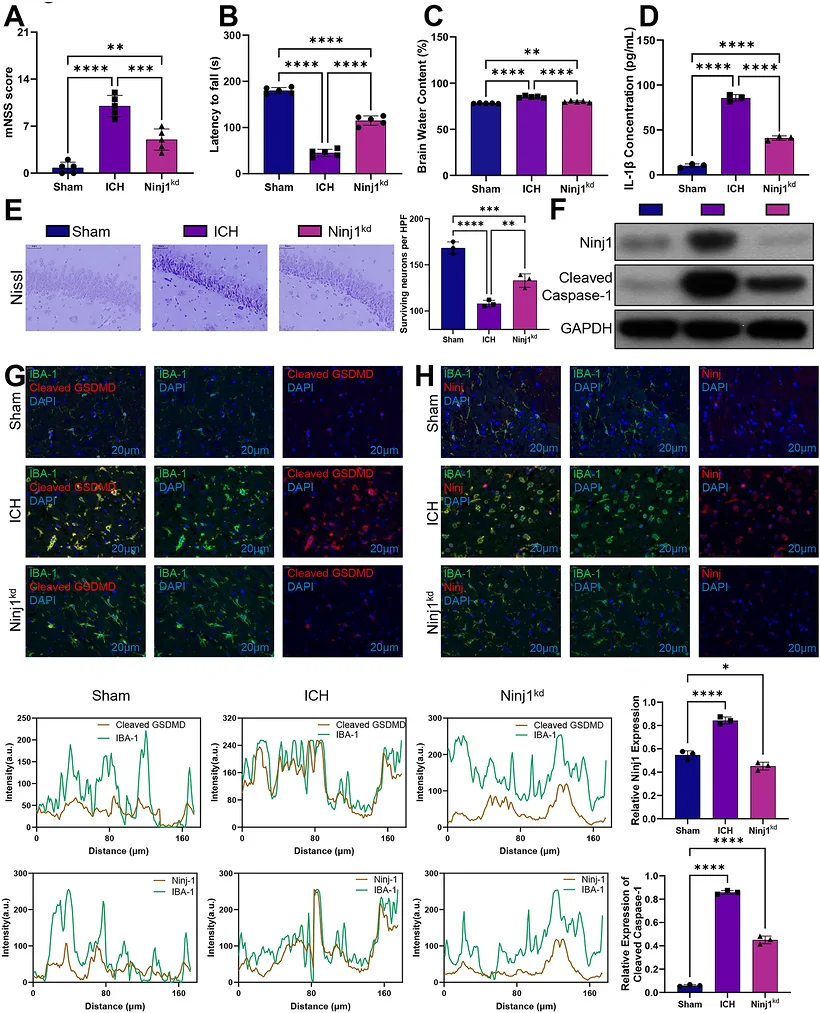
Effects of in vivo Ninj1 knockdown on neurological deficits and neuroinflammation after ICH. (A–C) mNSS (A), Rotarod latency (B), and brain water content (C) at 72 h post‐ICH (*n = *6 per group). (D) Tissue IL‐1β concentration by ELISA (*n = *3 per group). (E) Nissl‐stained sections and quantification of surviving neurons (*n = *3 per group). (F) Immunoblots and relative quantification of Ninj1 and Cleaved Caspase‐1 (*n = *3 per group). (G, H) Double immunofluorescence of IBA1 with Cleaved GSDMD (G) or Ninj1 (H), accompanied by fluorescence intensity profile scans (scale bars = 20 µm, *n = *3 per group). Values represent mean ± SD. For terminal biochemical and histological assays (D–H), tissue was partitioned from parallel cohorts (*n = *3 per group) to minimize animal usage. Statistical analysis was performed using one‐way ANOVA with Tukey's post‐hoc test (*p < 0.05, **p < 0.01, ***p < 0.001, ****p < 0.0001).

## 4 Discussion

As a severe neurological catastrophe, ICH is characterized by substantial morbidity and mortality, which are predominantly driven by hematoma‐induced SBI (Tsai et al. 2024). Throughout this pathological progression, dysregulated neuroinflammation serves as a central catalyst for cerebral edema and irreversible neuronal loss, culminating in enduring functional deficits (Cao et al. 2018). Given the current absence of targeted pharmacological interventions, patients frequently experience unfavorable clinical trajectories, generating substantial socioeconomic burdens (Xu et al. 2024). Consequently, elucidating novel molecular mediators within the neuroinflammatory cascade remains imperative for the advancement of neuroprotective strategies.

In an effort to identify candidate genes orchestrating this inflammatory response, our initial re‐analysis of public transcriptomic datasets highlighted the significant activation of pathways related to membrane remodeling and phagocytosis. Within this context, Ninj1 emerged as a prominently upregulated candidate transcript, prompting further experimental validation. Consistent with the in silico predictions, our in vivo murine ICH model confirmed a robust elevation of Ninj1 expression at 72 h post‐injury, with immunofluorescence demonstrating its predominant localization within activated (IBA1+) microglia. Extensive literature has established microglial activation as a hallmark of various neurodegenerative conditions and hemorrhagic stroke (Zhao et al. 2026; Sun et al. 2024). For instance, previous investigations have shown that therapeutic modulation of microglial phenotypes—such as neutralizing IL‐17A or silencing CXCL16—confers significant neuroprotection by curtailing deleterious inflammatory polarization (Yu et al. 2016; Dingyi et al. 2024). While our observations align with this established microglia‐centric pathological framework, the specific molecular events linking Ninj1 to the execution of microglial pyroptosis required systematic exploration.

Originally characterized as an adhesion molecule within the nervous system, Ninj1 has classically been associated with developmental and regenerative processes, such as facilitating Schwann cell migration following peripheral nerve trauma (Wu et al. 2024; Dun and Parkinson 2017). However, a paradigm shift in recent literature has uncovered its deleterious function in exacerbating sterile inflammation. Specifically, targeted deletion or inhibition of Ninj1 has proven protective against acute hepatic failure and renal injury by preventing cytolysis (Kim et al. 2022; Zhou et al. 2025). Mechanistically, Ninj1 is now recognized as the terminal executioner across multiple lytic cell death modalities, strictly required for the final disintegration of the plasma membrane (Ramos et al. 2024). Despite these advances, its direct involvement in microglial pyroptosis during ICH remained uncharacterized. Our in vitro investigations revealed that targeted silencing of Ninj1 in hemin‐challenged microglia robustly impeded the pyroptotic cascade. This intervention not only suppressed the cleavage of upstream mediators (Caspase‐1 and GSDMD) but also prevented plasma membrane rupture and the subsequent efflux of IL‐1β, thereby mitigating neurotoxic secondary effects on co‐cultured neurons.

Recognized as a highly pro‐inflammatory modality of programmed cell death, pyroptosis is propagated by a complex network of inflammatory cascades (Liu et al. 2023). Within the specific pathological microenvironment of ICH, the initiation of this lytic process is classically defined by the assembly of the canonical NLRP3 inflammasome and the subsequent engagement of its downstream effectors (Gu et al. 2022). Previous investigations have highlighted the therapeutic potential of targeting this axis; for instance, suppressing the cathepsin B/NLRP3 signaling cascade via Cystatin C provides robust neuroprotection in the perihematomal region (Zhou et al. 2024). Accordingly, the application of selective pharmacological inhibitors directed against the NLRP3 complex consistently mitigates post‐hemorrhagic brain injury (Ren et al. 2018). Upstream of this inflammasome assembly, the NF‐κB cascade functions as the critical “Signal 1,” orchestrating the primary transcriptional activation of numerous pro‐inflammatory mediators, including NLRP3, thereby serving as a pivotal node for alleviating ICH‐induced injury (Yu et al. 2025). Despite a comprehensive understanding of these upstream initiation events, the precise mechanisms governing the ultimate physical rupture of the plasma membrane and the massive extrusion of intracellular contents have remained a critical unresolved paradigm. Our in vitro analyses indicated that the NF‐κB transcription factor promotes the expression of both NLRP3 and Ninj1. Although direct promoter enrichment was statistically significant only for Nlrp3 in our ChIP‐qPCR assay, dual‐luciferase and EMSA validations support the transcriptional activation potential of p65 on both promoters, suggesting a coordinated and potentially indirect regulatory network regulating the microglial pyroptotic program. This coordinated signaling facilitates the proteolytic maturation of GSDMD and culminates in Ninj1‐dependent membrane disintegration, thereby driving secondary neuronal toxicity. Consistent with these mechanistic insights, targeted in vivo silencing of this terminal executioner significantly blunted the neuroinflammatory response, preserved perihematomal neuronal viability, and improved overall neurological recovery following ICH.

Furthermore, our rescue experiments support the hypothesis of a positive feedback linking Ninj1 to upstream inflammasome activation. Although classically recognized as a downstream pyroptotic executioner, Ninj1 knockdown concurrently reduced the levels of upstream cleaved caspase‐1 and GSDMD. Conversely, restoring Ninj1 expression (oe‐Ninj1) effectively re‐activated these proteins (Figure S1). This phenomenon is likely driven by the restriction of damage‐associated molecular pattern (DAMP) release. By preventing Ninj1‐dependent membrane rupture, the extracellular leakage of DAMPs, notably HMGB1, is effectively restricted, which aligns with recent observations in a spinal cord injury model (Yao et al. 2026). Once in the extracellular space, HMGB1 is known to activate the NLRP3 inflammasome in microglia and exacerbate neuroinflammation, including in ICH (Liu et al. 2022, Lei et al. 2024). Therefore, interrupting this autocrine/paracrine amplification loop via Ninj1 depletion dampens the sustained hyperactivation of NLRP3. These findings highlight that Ninj1‐mediated membrane rupture is not merely a terminal event in cell death, but serves as a critical amplifier of the neuroinflammatory cascade.

In conclusion, the present investigation delineates the critical contribution of the NF‐κB/Ninj1 signaling cascade to secondary neural injury following ICH. Utilizing complementary in vivo murine models and in vitro paradigms, our findings indicate that the NF‐κB transcription factor regulates the expression of both NLRP3 and Ninj1 to modulate the microglial pyroptotic program, although the physical promoter‐binding dynamics of p65 vary between these target promoters. Despite these mechanistic insights, several limitations warrant acknowledgment. First, intracerebroventricular siRNA administration and IBA1 immunostaining lack absolute cell‐type specificity, rendering the in vivo neuroprotection a general myeloid‐lineage validation rather than microglia‐exclusive silencing. Second, our observations are constrained to the acute pathological window (72 h) post‐hemorrhage; the temporal dynamics of Ninj1 during chronic inflammation, long‐term tissue remodeling, and neural repair remain to be fully characterized. Third, although the immortalized BV‐2 and HT22 cell lines provide robust platforms for initial mechanistic screening, they may not perfectly mirror the intricate biological phenotypes of native primary cells. Consequently, incorporating primary cellular cultures and advanced translational models in future studies will be imperative to substantiate the physiological relevance of this pathway. Finally, owing to the inherent ethical and logistical constraints associated with acquiring perihematomal clinical specimens, our findings lack direct validation in human ICH tissue. As such, the translational potential of targeting Ninj1‐dependent pyroptosis must be interpreted with caution until corroborated by human pathological data. Ultimately, while this study unveils a novel regulatory node within the post‐ICH inflammatory network, rigorous translational research is required before Ninj1 can be established as a viable clinical target.

## Author Contributions

**Liqiang Peng**: investigation, methodology, visualization, writing – original draft, funding acquisition, writing – review and editing. **Aosheng Peng**: data curation, funding acquisition, methodology, writing – original draft, writing – review and editing. **Hui Zhu**: data curation, formal analysis, investigation, funding acquisition, writing – review and editing. **Ting Qi**: data curation, validation, funding acquisition, writing – review and editing. **Ting Sun**: conceptualization, supervision, funding acquisition, writing – review and editing. **Pan Liu**: conceptualization, supervision, funding acquisition, project administration, writing – review and editing.

## Funding

This study was supported by the Hunan Natural Science Foundation (2024JJ7663).

## Ethics Statement

The study protocol for animal experiments was reviewed and approved by the Experimental Animal Ethics Committee of Zhuzhou Hospital Affiliated to Xiangya School of Medicine (Approval No. K2025‐01‐223‐412). All animal experiments were conducted in strict accordance with the institutional guidelines for the care and use of laboratory animals of Zhuzhou Hospital Affiliated to Xiangya School of Medicine, ensuring the ethical treatment and welfare of all animals involved. Furthermore, all methods involving animals were reported in accordance with the ARRIVE guidelines.

## Conflicts of Interest

The authors declare no conflicts of interest.

## Supporting information




**Supplementary Material**: brb371862‐sup‐0001‐SuppMat.docx

## Data Availability

Data are available from the corresponding author on reasonable request.
