# Supplementary material for: Targeting Ninjurin‐1‐Mediated Microglial Pyroptosis Attenuates Neuroinflammation and Neuronal Injury Following Intracerebral Hemorrhage
Source: Brain Behav. 2026 Nov 1;16(11):e71862. doi: 10.1002/brb3.71862 (PMC13724796; doi:10.1002/brb3.71862)
Supplement: Supplementary file 1 [file BRB3-16-e71862-s001.docx]

**Targeting Ninjurin-1-Mediated Microglial Pyroptosis Attenuates Neuroinflammation and Neuronal Injury Following Intracerebral Hemorrhage**

Liqiang Peng^1, #^, Aosheng Peng^2, #^, Hui Zhu^2, #^, Ting Sun^2, *^, Pan Liu^3, *^, Ting Qi^4^

^1^Department of Trauma Center, Zhuzhou Hospital Affiliated to Xiangya School of Medicine, Central South University, Zhuzhou City,Hunan Province,PR China

^2^Department of Emergency, Zhuzhou Hospital Affiliated to Xiangya School of Medicine, Central South University, Zhuzhou City, Hunan Province, PR China

^3^Health Management Center, Zhuzhou Hospital Affiliated to Xiangya School of Medicine, Central South University, Zhuzhou City, Hunan Province, PR China

^4^Department of General Surgery, Zhuzhou Hospital Affiliated to Xiangya School of Medicine, Central South University, Zhuzhou City, Hunan Province, PR China

^#^ These authors contributed equally to this work.

**Catalogue**

**Figure S1**…………Restoring Ninj1 reverses the suppression of upstream pyroptotic mediators in Hemin-challenged microglia.

**Figure S2**…………Validation of Ninj1-driven pyroptotic signaling in primary murine microglia.

**Figure S3**…………Single-cell transcriptomic profiling of Ninj1 expression in the brain post-ICH.

**Figure S4**…………Uncropped immunoblot scans supporting the representative data.

**Figure S1. Restoring Ninj1 reverses the suppression of upstream pyroptotic mediators in Hemin-challenged microglia.** BV-2 cells underwent co-transfection with sh-Ninj1 and a Ninj1-overexpressing plasmid (oe-Ninj1) prior to Hemin exposure. The protein expressions of Ninj1, Cleaved Caspase-1, Cleaved GSDMD, and Total GSDMD were evaluated utilizing immunoblot analysis, with GAPDH serving as the endogenous control. Densitometric quantification is displayed in the accompanying bar charts. Data represent the mean ± SD (n = 3 independent experiments).

**Figure S2. Validation of Ninj1-driven pyroptotic signaling in primary murine microglia.** Primary microglial cultures were transfected with sh-Ninj1 and subsequently subjected to Hemin stimulation. Immunoblotting was conducted to determine the specific protein levels of Ninj1, along with the cleavage of Caspase-1 and GSDMD. GAPDH was utilized to ensure equal protein loading. Quantitative densitometry results are summarized in the bar graphs. Data are expressed as mean ± SD (n = 3 independent experiments).

**Figure S3.** Single-cell transcriptomic profiling of Ninj1 expression in the brain post-ICH. (A) UMAP plot showing cellular annotations of reconstructed clusters within the hemorrhagic microenvironment. (B) Feature plot showing the distribution of Ninj1 transcripts, demonstrating enrichment within myeloid-lineage clusters. (C) Violin plot representing Ninj1 transcript levels across cell types. The expression is enriched in myeloid-lineage cells, exhibiting prominent levels in neutrophils, activated macrophages, and microglia/macrophages, while remaining negligible in non-myeloid resident central nervous system cells such as astrocytes and neurons.

Figure S4. Uncropped immunoblot scans supporting the representative data. This figure presents the full-length, unedited membrane images corresponding to the cropped protein bands displayed throughout the main manuscript and preceding supplementary figures. Standard molecular weight markers are explicitly annotated alongside each blot to validate the specific target sizes.

Figure 2B

Figure 3A-B

Figure 4B

Figure 5B

Figure 5B

Figure 6E

Figure 7F

Supporting information Figure S1

Supporting information Figure S2
